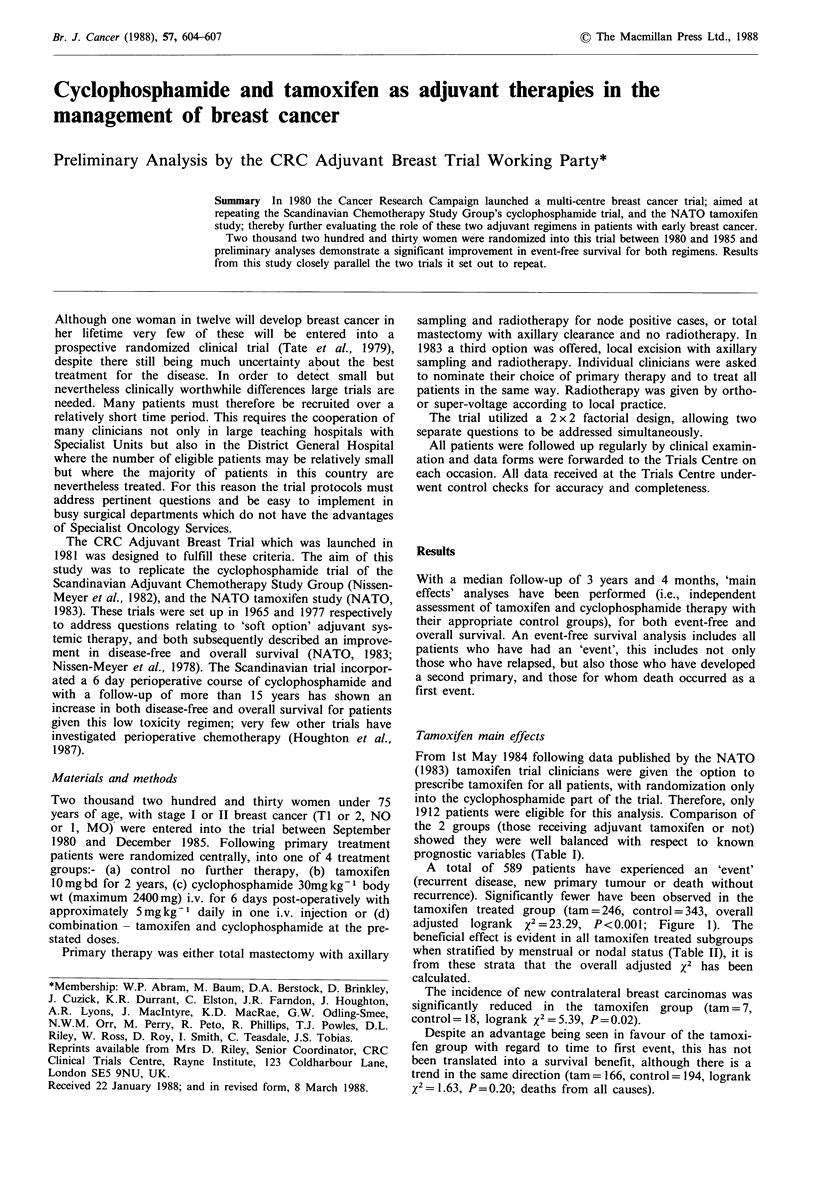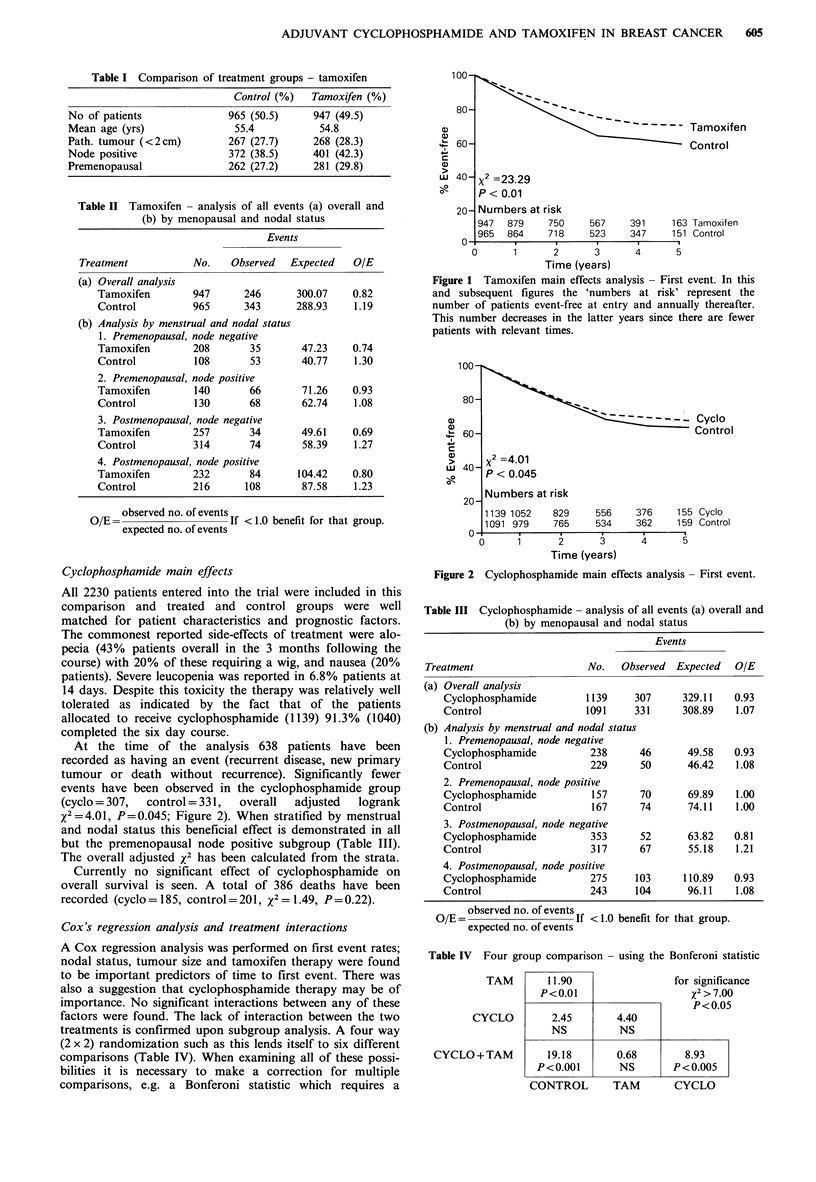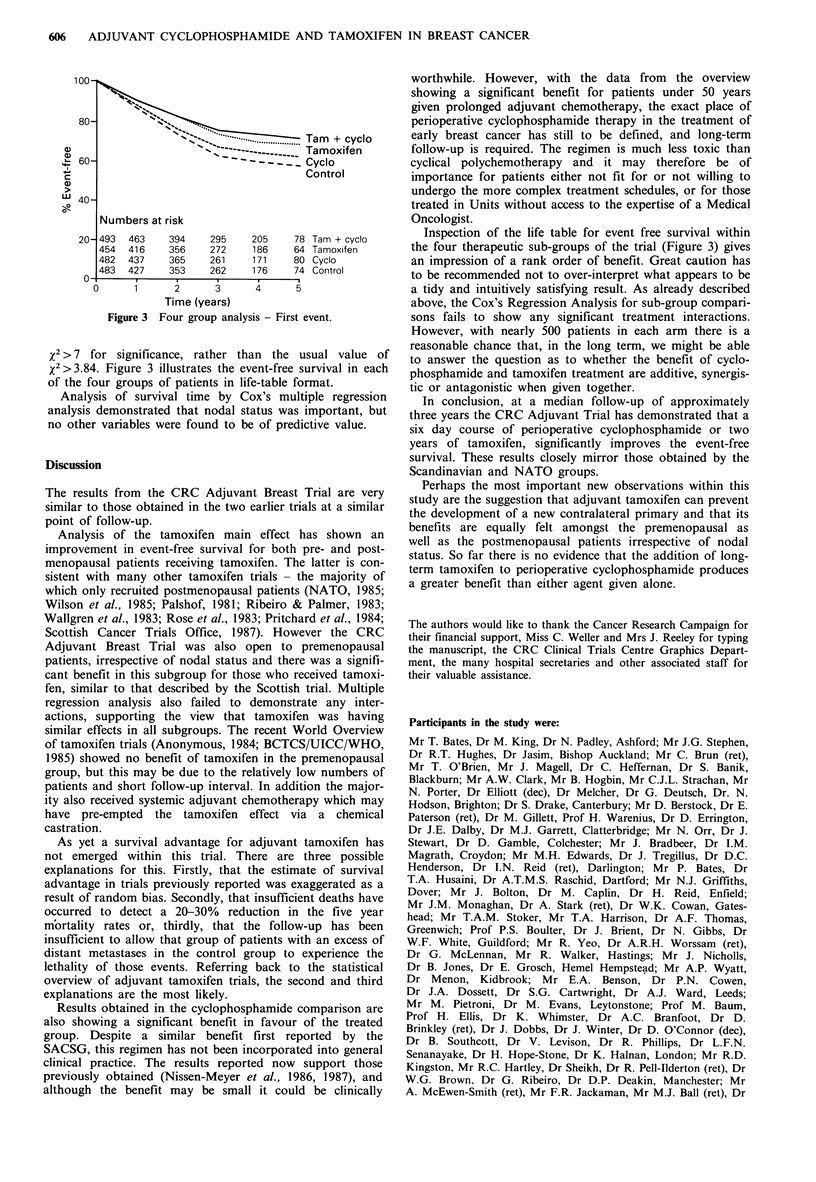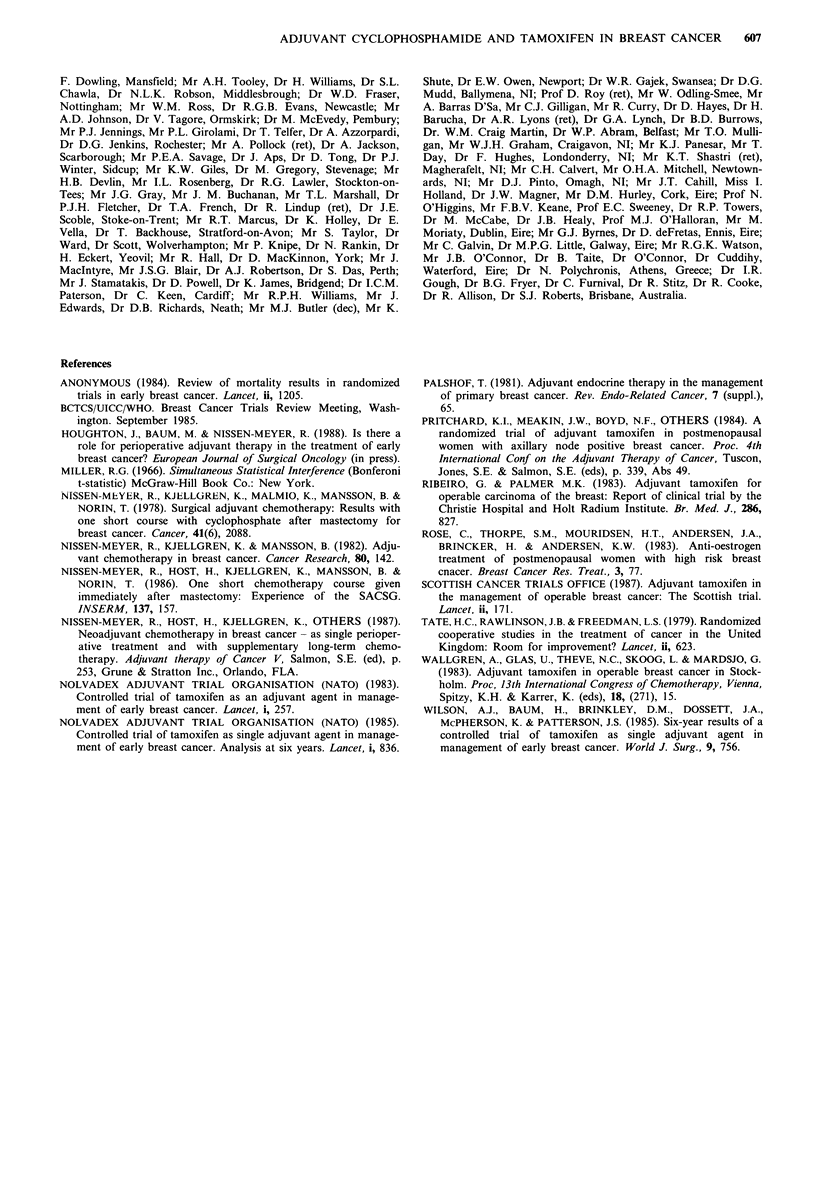# Cyclophosphamide and tamoxifen as adjuvant therapies in the management of breast cancer. CRC Adjuvant Breast Trial Working Party.

**DOI:** 10.1038/bjc.1988.137

**Published:** 1988-06

**Authors:** 

## Abstract

In 1980 the Cancer Research Campaign launched a multi-centre breast cancer trial; aimed at repeating the Scandinavian Chemotherapy Study Group's cyclophosphamide trial, and the NATO tamoxifen study; thereby further evaluating the role of these two adjuvant regimens in patients with early breast cancer. Two thousand two hundred and thirty women were randomized into this trial between 1980 and 1985 and preliminary analyses demonstrate a significant improvement in event-free survival for both regimens. Results from this study closely parallel the two trials it set out to repeat.


Br. J. Cancer (1988), 57, 604–607 © The Macmillan Press Ltd., 1988

### Cyclophosphamide and tamoxifen as adjuvant therapies in the management of breast cancer

Preliminary Analysis by the CRC Adjuvant Breast Trial Working Party*

**Summary** In 1980 the Cancer Research Campaign launched a multi-centre breast cancer trial; aimed at repeating the Scandinavian Chemotherapy Study Group's cyclophosphamide trial, and the NATO tamoxifen study; thereby further evaluating the role of these two adjuvant regimens in patients with early breast cancer. Two thousand two hundred and thirty women were randomized into this trial between 1980 and 1985 and preliminary analyses demonstrate a significant improvement in event-free survival for both regimens. Results from this study closely parallel the two trials it set out to repeat.

Although one woman in twelve will develop breast cancer in her lifetime very few of these will be entered into a prospective randomized clinical trial (Tate *et al.*, 1979), despite there still being much uncertainty about the best treatment for the disease. In order to detect small but nevertheless clinically worthwhile differences large trials are needed. Many patients must therefore be recruited over a relatively short time period. This requires the cooperation of many clinicians not only in large teaching hospitals with Specialist Units but also in the District General Hospital where the number of eligible patients may be relatively small but where the majority of patients in this country are nevertheless treated. For this reason the trial protocols must address pertinent questions and be easy to implement in busy surgical departments which do not have the advantages of Specialist Oncology Services.

The CRC Adjuvant Breast Trial which was launched in 1981 was designed to fulfill these criteria. The aim of this study was to replicate the cyclophosphamide trial of the Scandinavian Adjuvant Chemotherapy Study Group (Nissen-Meyer *et al.*, 1982), and the NATO tamoxifen study (NATO, 1983). These trials were set up in 1965 and 1977 respectively to address questions relating to 'soft option' adjuvant systemic therapy, and both subsequently described an improvement in disease-free and overall survival (NATO, 1983; Nissen-Meyer *et al.*, 1978). The Scandinavian trial incorporated a 6 day perioperative course of cyclophosphamide and with a follow-up of more than 15 years has shown an increase in both disease-free and overall survival for patients given this low toxicity regimen; very few other trials have investigated perioperative chemotherapy (Houghton *et al.*, 1987).

### *Materials and methods*

Two thousand two hundred and thirty women under 75 years of age, with stage I or II breast cancer (T1 or 2, NO or 1, MO) were entered into the trial between September 1980 and December 1985. Following primary treatment patients were randomized centrally, into one of 4 treatment groups:- (a) control no further therapy, (b) tamoxifen 10 mg bd for 2 years, (c) cyclophosphamide 30mg $kg^{-1}$ body wt (maximum 2400 mg) i.v. for 6 days post-operatively with approximately 5 mg $kg^{-1}$ daily in one i.v. injection or (d) combination – tamoxifen and cyclophosphamide at the pre-stated doses.

Primary therapy was either total mastectomy with axillary sampling and radiotherapy for node positive cases, or total mastectomy with axillary clearance and no radiotherapy. In 1983 a third option was offered, local excision with axillary sampling and radiotherapy. Individual clinicians were asked to nominate their choice of primary therapy and to treat all patients in the same way. Radiotherapy was given by ortho- or super-voltage according to local practice.

The trial utilized a $2 \times 2$ factorial design, allowing two separate questions to be addressed simultaneously.

All patients were followed up regularly by clinical examination and data forms were forwarded to the Trials Centre on each occasion. All data received at the Trials Centre underwent control checks for accuracy and completeness.

### Results

With a median follow-up of 3 years and 4 months, 'main effects' analyses have been performed (i.e., independent assessment of tamoxifen and cyclophosphamide therapy with their appropriate control groups), for both event-free and overall survival. An event-free survival analysis includes all patients who have had an 'event', this includes not only those who have relapsed, but also those who have developed a second primary, and those for whom death occurred as a first event.

### *Tamoxifen main effects*

From 1st May 1984 following data published by the NATO (1983) tamoxifen trial clinicians were given the option to prescribe tamoxifen for all patients, with randomization only into the cyclophosphamide part of the trial. Therefore, only 1912 patients were eligible for this analysis. Comparison of the 2 groups (those receiving adjuvant tamoxifen or not) showed they were well balanced with respect to known prognostic variables (Table I).

A total of 589 patients have experienced an 'event' (recurrent disease, new primary tumour or death without recurrence). Significantly fewer have been observed in the tamoxifen treated group (tam = 246, control = 343, overall adjusted logrank $\chi^2 = 23.29$, $P < 0.001$; Figure 1). The beneficial effect is evident in all tamoxifen treated subgroups when stratified by menstrual or nodal status (Table II), it is from these strata that the overall adjusted $\chi^2$ has been calculated.

The incidence of new contralateral breast carcinomas was significantly reduced in the tamoxifen group (tam = 7, control = 18, logrank $\chi^2 = 5.39$, $P = 0.02$).

Despite an advantage being seen in favour of the tamoxifen group with regard to time to first event, this has not been translated into a survival benefit, although there is a trend in the same direction (tam = 166, control = 194, logrank $\chi^2 = 1.63$, $P = 0.20$; deaths from all causes).

*Membership: W.P. Abram, M. Baum, D.A. Berstock, D. Brinkley, J. Cuzick, K.R. Durrant, C. Elston, J.R. Farndon, J. Houghton, A.R. Lyons, J. MacIntyre, K.D. MacRae, G.W. Odling-Smee, N.W.M. Orr, M. Perry, R. Peto, R. Phillips, T.J. Powles, D.L. Riley, W. Ross, D. Roy, I. Smith, C. Teasdale, J.S. Tobias.
Reprints available from Mrs D. Riley, Senior Coordinator, CRC Clinical Trials Centre, Rayne Institute, 123 Coldharbour Lane, London SE5 9NU, UK.
Received 22 January 1988; and in revised form, 8 March 1988.

**Table I** Comparison of treatment groups – tamoxifen

| | *Control* (%) | *Tamoxifen* (%) |
|---|---|---|
| No of patients | 965 (50.5) | 947 (49.5) |
| Mean age (yrs) | 55.4 | 54.8 |
| Path. tumour (<2 cm) | 267 (27.7) | 268 (28.3) |
| Node positive | 372 (38.5) | 401 (42.3) |
| Premenopausal | 262 (27.2) | 281 (29.8) |

**Table II** Tamoxifen – analysis of all events (a) overall and (b) by menopausal and nodal status

| | | *Events* | | |
|---|---|---|---|---|
| *Treatment* | *No.* | *Observed* | *Expected* | *O/E* |
| (a) *Overall analysis* | | | | |
| Tamoxifen | 947 | 246 | 300.07 | 0.82 |
| Control | 965 | 343 | 288.93 | 1.19 |
| (b) *Analysis by menstrual and nodal status* | | | | |
| 1. *Premenopausal, node negative* | | | | |
| Tamoxifen | 208 | 35 | 47.23 | 0.74 |
| Control | 108 | 53 | 40.77 | 1.30 |
| 2. *Premenopausal, node positive* | | | | |
| Tamoxifen | 140 | 66 | 71.26 | 0.93 |
| Control | 130 | 68 | 62.74 | 1.08 |
| 3. *Postmenopausal, node negative* | | | | |
| Tamoxifen | 257 | 34 | 49.61 | 0.69 |
| Control | 314 | 74 | 58.39 | 1.27 |
| 4. *Postmenopausal, node positive* | | | | |
| Tamoxifen | 232 | 84 | 104.42 | 0.80 |
| Control | 216 | 108 | 87.58 | 1.23 |

$O/E = \frac{\text{observed no. of events}}{\text{expected no. of events}}$ If <1.0 benefit for that group.

### *Cyclophosphamide main effects*

All 2230 patients entered into the trial were included in this comparison and treated and control groups were well matched for patient characteristics and prognostic factors. The commonest reported side-effects of treatment were alopecia (43% patients overall in the 3 months following the course) with 20% of these requiring a wig, and nausea (20% patients). Severe leucopenia was reported in 6.8% patients at 14 days. Despite this toxicity the therapy was relatively well tolerated as indicated by the fact that of the patients allocated to receive cyclophosphamide (1139) 91.3% (1040) completed the six day course.

At the time of the analysis 638 patients have been recorded as having an event (recurrent disease, new primary tumour or death without recurrence). Significantly fewer events have been observed in the cyclophosphamide group (cyclo = 307, control = 331, overall adjusted logrank $\chi^2 = 4.01$, $P = 0.045$; Figure 2). When stratified by menstrual and nodal status this beneficial effect is demonstrated in all but the premenopausal node positive subgroup (Table III). The overall adjusted $\chi^2$ has been calculated from the strata.

Currently no significant effect of cyclophosphamide on overall survival is seen. A total of 386 deaths have been recorded (cyclo = 185, control = 201, $\chi^2 = 1.49$, $P = 0.22$).

### *Cox's regression analysis and treatment interactions*

A Cox regression analysis was performed on first event rates; nodal status, tumour size and tamoxifen therapy were found to be important predictors of time to first event. There was also a suggestion that cyclophosphamide therapy may be of importance. No significant interactions between any of these factors were found. The lack of interaction between the two treatments is confirmed upon subgroup analysis. A four way (2 x 2) randomization such as this lends itself to six different comparisons (Table IV). When examining all of these possibilities it is necessary to make a correction for multiple comparisons, e.g. a Bonferoni statistic which requires a

**Figure 1** Tamoxifen main effects analysis – First event. In this and subsequent figures the 'numbers at risk' represent the number of patients event-free at entry and annually thereafter. This number decreases in the latter years since there are fewer patients with relevant times.

**Figure 2** Cyclophosphamide main effects analysis – First event.

**Table III** Cyclophosphamide – analysis of all events (a) overall and (b) by menopausal and nodal status

| | | *Events* | | |
|---|---|---|---|---|
| *Treatment* | *No.* | *Observed* | *Expected* | *O/E* |
| (a) *Overall analysis* | | | | |
| Cyclophosphamide | 1139 | 307 | 329.11 | 0.93 |
| Control | 1091 | 331 | 308.89 | 1.07 |
| (b) *Analysis by menstrual and nodal status* | | | | |
| 1. *Premenopausal, node negative* | | | | |
| Cyclophosphamide | 238 | 46 | 49.58 | 0.93 |
| Control | 229 | 50 | 46.42 | 1.08 |
| 2. *Premenopausal, node positive* | | | | |
| Cyclophosphamide | 157 | 70 | 69.89 | 1.00 |
| Control | 167 | 74 | 74.11 | 1.00 |
| 3. *Postmenopausal, node negative* | | | | |
| Cyclophosphamide | 353 | 52 | 63.82 | 0.81 |
| Control | 317 | 67 | 55.18 | 1.21 |
| 4. *Postmenopausal, node positive* | | | | |
| Cyclophosphamide | 275 | 103 | 110.89 | 0.93 |
| Control | 243 | 104 | 96.11 | 1.08 |

$O/E = \frac{\text{observed no. of events}}{\text{expected no. of events}}$ If <1.0 benefit for that group.

**Table IV** Four group comparison – using the Bonferoni statistic

| | | | |
|---|---|---|---|
| TAM | 11.90 $P<0.01$ | | for significance $\chi^2>7.00$ $P<0.05$ |
| CYCLO | 2.45 NS | 4.40 NS | |
| CYCLO+TAM | 19.18 $P<0.001$ | 0.68 NS | 8.93 $P<0.005$ |
| | CONTROL | TAM | CYCLO |

**Figure 3** Four group analysis – First event.

$\chi^2 > 7$ for significance, rather than the usual value of $\chi^2 > 3.84$. Figure 3 illustrates the event-free survival in each of the four groups of patients in life-table format.

Analysis of survival time by Cox's multiple regression analysis demonstrated that nodal status was important, but no other variables were found to be of predictive value.

### Discussion

The results from the CRC Adjuvant Breast Trial are very similar to those obtained in the two earlier trials at a similar point of follow-up.

Analysis of the tamoxifen main effect has shown an improvement in event-free survival for both pre- and post-menopausal patients receiving tamoxifen. The latter is consistent with many other tamoxifen trials – the majority of which only recruited postmenopausal patients (NATO, 1985; Wilson *et al.*, 1985; Palshof, 1981; Ribeiro & Palmer, 1983; Wallgren *et al.*, 1983; Rose *et al.*, 1983; Pritchard *et al.*, 1984; Scottish Cancer Trials Office, 1987). However the CRC Adjuvant Breast Trial was also open to premenopausal patients, irrespective of nodal status and there was a significant benefit in this subgroup for those who received tamoxifen, similar to that described by the Scottish trial. Multiple regression analysis also failed to demonstrate any interactions, supporting the view that tamoxifen was having similar effects in all subgroups. The recent World Overview of tamoxifen trials (Anonymous, 1984; BCTCS/UICC/WHO, 1985) showed no benefit of tamoxifen in the premenopausal group, but this may be due to the relatively low numbers of patients and short follow-up interval. In addition the majority also received systemic adjuvant chemotherapy which may have pre-empted the tamoxifen effect via a chemical castration.

As yet a survival advantage for adjuvant tamoxifen has not emerged within this trial. There are three possible explanations for this. Firstly, that the estimate of survival advantage in trials previously reported was exaggerated as a result of random bias. Secondly, that insufficient deaths have occurred to detect a 20–30% reduction in the five year mortality rates or, thirdly, that the follow-up has been insufficient to allow that group of patients with an excess of distant metastases in the control group to experience the lethality of those events. Referring back to the statistical overview of adjuvant tamoxifen trials, the second and third explanations are the most likely.

Results obtained in the cyclophosphamide comparison are also showing a significant benefit in favour of the treated group. Despite a similar benefit first reported by the SACSG, this regimen has not been incorporated into general clinical practice. The results reported now support those previously obtained (Nissen-Meyer *et al.*, 1986, 1987), and although the benefit may be small it could be clinically worthwhile. However, with the data from the overview showing a significant benefit for patients under 50 years given prolonged adjuvant chemotherapy, the exact place of perioperative cyclophosphamide therapy in the treatment of early breast cancer has still to be defined, and long-term follow-up is required. The regimen is much less toxic than cyclical polychemotherapy and it may therefore be of importance for patients either not fit for or not willing to undergo the more complex treatment schedules, or for those treated in Units without access to the expertise of a Medical Oncologist.

Inspection of the life table for event free survival within the four therapeutic sub-groups of the trial (Figure 3) gives an impression of a rank order of benefit. Great caution has to be recommended not to over-interpret what appears to be a tidy and intuitively satisfying result. As already described above, the Cox's Regression Analysis for sub-group comparisons fails to show any significant treatment interactions. However, with nearly 500 patients in each arm there is a reasonable chance that, in the long term, we might be able to answer the question as to whether the benefit of cyclophosphamide and tamoxifen treatment are additive, synergistic or antagonistic when given together.

In conclusion, at a median follow-up of approximately three years the CRC Adjuvant Trial has demonstrated that a six day course of perioperative cyclophosphamide or two years of tamoxifen, significantly improves the event-free survival. These results closely mirror those obtained by the Scandinavian and NATO groups.

Perhaps the most important new observations within this study are the suggestion that adjuvant tamoxifen can prevent the development of a new contralateral primary and that its benefits are equally felt amongst the premenopausal as well as the postmenopausal patients irrespective of nodal status. So far there is no evidence that the addition of long-term tamoxifen to perioperative cyclophosphamide produces a greater benefit than either agent given alone.

The authors would like to thank the Cancer Research Campaign for their financial support, Miss C. Weller and Mrs J. Reeley for typing the manuscript, the CRC Clinical Trials Centre Graphics Department, the many hospital secretaries and other associated staff for their valuable assistance.

**Participants in the study were:**

Mr T. Bates, Dr M. King, Dr N. Padley, Ashford; Mr J.G. Stephen, Dr R.T. Hughes, Dr Jasim, Bishop Auckland; Mr C. Brun (ret), Mr T. O'Brien, Mr J. Magell, Dr C. Heffernan, Dr S. Banik, Blackburn; Mr A.W. Clark, Mr B. Hogbin, Mr C.J.L. Strachan, Mr N. Porter, Dr Elliott (dec), Dr Melcher, Dr G. Deutsch, Dr. N. Hodson, Brighton; Dr S. Drake, Canterbury; Mr D. Berstock, Dr E. Paterson (ret), Dr M. Gillett, Prof H. Warenius, Dr D. Errington, Dr J.E. Dalby, Dr M.J. Garrett, Clatterbridge; Mr N. Orr, Dr J. Stewart, Dr D. Gamble, Colchester; Mr J. Bradbeer, Dr I.M. Magrath, Croydon; Mr M.H. Edwards, Dr J. Tregillus, Dr D.C. Henderson, Dr I.N. Reid (ret), Darlington; Mr P. Bates, Dr T.A. Husaini, Dr A.T.M.S. Raschid, Dartford; Mr N.J. Griffiths, Dover; Mr J. Bolton, Dr M. Caplin, Dr H. Reid, Enfield; Mr J.M. Monaghan, Dr A. Stark (ret), Dr W.K. Cowan, Gateshead; Mr T.A.M. Stoker, Mr T.A. Harrison, Dr A.F. Thomas, Greenwich; Prof P.S. Boulter, Dr J. Brient, Dr N. Gibbs, Dr W.F. White, Guildford; Mr R. Yeo, Dr A.R.H. Worssam (ret), Dr G. McLennan, Mr R. Walker, Hastings; Mr J. Nicholls, Dr B. Jones, Dr E. Grosch, Hemel Hempstead; Mr A.P. Wyatt, Dr Menon, Kidbrook; Mr E.A. Benson, Dr P.N. Cowen, Dr J.A. Dossett, Dr S.G. Cartwright, Dr A.J. Ward, Leeds; Mr M. Pietroni, Dr M. Evans, Leytonstone; Prof M. Baum, Prof H. Ellis, Dr K. Whimster, Dr A.C. Branfoot, Dr D. Brinkley (ret), Dr J. Dobbs, Dr J. Winter, Dr D. O'Connor (dec), Dr B. Southcott, Dr V. Levison, Dr R. Phillips, Dr L.F.N. Senanayake, Dr H. Hope-Stone, Dr K. Halnan, London; Mr R.D. Kingston, Mr R.C. Hartley, Dr Sheikh, Dr R. Pell-Ilderton (ret), Dr W.G. Brown, Dr G. Ribeiro, Dr D.P. Deakin, Manchester; Mr A. McEwen-Smith (ret), Mr F.R. Jackaman, Mr M.J. Ball (ret), Dr

F. Dowling, Mansfield; Mr A.H. Tooley, Dr H. Williams, Dr S.L. Chawla, Dr N.L.K. Robson, Middlesbrough; Dr W.D. Fraser, Nottingham; Mr W.M. Ross, Dr R.G.B. Evans, Newcastle; Mr A.D. Johnson, Dr V. Tagore, Ormskirk; Dr M. McEvedy, Pembury; Mr P.J. Jennings, Mr P.L. Girolami, Dr T. Telfer, Dr A. Azzorpardi, Dr D.G. Jenkins, Rochester; Mr A. Pollock (ret), Dr A. Jackson, Scarborough; Mr P.E.A. Savage, Dr J. Aps, Dr D. Tong, Dr P.J. Winter, Sidcup; Mr K.W. Giles, Dr M. Gregory, Stevenage; Mr H.B. Devlin, Mr I.L. Rosenberg, Dr R.G. Lawler, Stockton-on-Tees; Mr J.G. Gray, Mr J. M. Buchanan, Mr T.L. Marshall, Dr P.J.H. Fletcher, Dr T.A. French, Dr R. Lindup (ret), Dr J.E. Scoble, Stoke-on-Trent; Mr R.T. Marcus, Dr K. Holley, Dr E. Vella, Dr T. Backhouse, Stratford-on-Avon; Mr S. Taylor, Dr Ward, Dr Scott, Wolverhampton; Mr P. Knipe, Dr N. Rankin, Dr H. Eckert, Yeovil; Mr R. Hall, Dr D. MacKinnon, York; Mr J. MacIntyre, Mr J.S.G. Blair, Dr A.J. Robertson, Dr S. Das, Perth; Mr J. Stamatakis, Dr D. Powell, Dr K. James, Bridgend; Dr I.C.M. Paterson, Dr C. Keen, Cardiff; Mr R.P.H. Williams, Mr J. Edwards, Dr D.B. Richards, Neath; Mr M.J. Butler (dec), Mr K. Shute, Dr E.W. Owen, Newport; Dr W.R. Gajek, Swansea; Dr D.G. Mudd, Ballymena, NI; Prof D. Roy (ret), Mr W. Odling-Smee, Mr A. Barras D'Sa, Mr C.J. Gilligan, Mr R. Curry, Dr D. Hayes, Dr H. Barucha, Dr A.R. Lyons (ret), Dr G.A. Lynch, Dr B.D. Burrows, Dr. W.M. Craig Martin, Dr W.P. Abram, Belfast; Mr T.O. Mulligan, Mr W.J.H. Graham, Craigavon, NI; Mr K.J. Panesar, Mr T. Day, Dr F. Hughes, Londonderry, NI; Mr K.T. Shastri (ret), Magherafelt, NI; Mr C.H. Calvert, Mr O.H.A. Mitchell, Newtownards, NI; Mr D.J. Pinto, Omagh, NI; Mr J.T. Cahill, Miss I. Holland, Dr J.W. Magner, Mr D.M. Hurley, Cork, Eire; Prof N. O'Higgins, Mr F.B.V. Keane, Prof E.C. Sweeney, Dr R.P. Towers, Dr M. McCabe, Dr J.B. Healy, Prof M.J. O'Halloran, Mr M. Moriaty, Dublin, Eire; Mr G.J. Byrnes, Dr D. deFretas, Ennis, Eire; Mr C. Galvin, Dr M.P.G. Little, Galway, Eire; Mr R.G.K. Watson, Mr J.B. O'Connor, Dr B. Taite, Dr O'Connor, Dr Cuddihy, Waterford, Eire; Dr N. Polychronis, Athens, Greece; Dr I.R. Gough, Dr B.G. Fryer, Dr C. Furnival, Dr R. Stitz, Dr R. Cooke, Dr R. Allison, Dr S.J. Roberts, Brisbane, Australia.

### References

ANONYMOUS (1984). Review of mortality results in randomized trials in early breast cancer. *Lancet*, **ii**, 1205.

BCTCS/UICC/WHO. Breast Cancer Trials Review Meeting, Washington. September 1985.

HOUGHTON, J., BAUM, M. & NISSEN-MEYER, R. (1988). Is there a role for perioperative adjuvant therapy in the treatment of early breast cancer? *European Journal of Surgical Oncology* (in press).

MILLER, R.G. (1966). *Simultaneous Statistical Interference* (Bonferoni t-statistic) McGraw-Hill Book Co.: New York.

NISSEN-MEYER, R., KJELLGREN, K., MALMIO, K., MANSSON, B. & NORIN, T. (1978). Surgical adjuvant chemotherapy: Results with one short course with cyclophosphate after mastectomy for breast cancer. *Cancer*, **41**(6), 2088.

NISSEN-MEYER, R., KJELLGREN, K. & MANSSON, B. (1982). Adjuvant chemotherapy in breast cancer. *Cancer Research*, **80**, 142.

NISSEN-MEYER, R., HOST, H., KJELLGREN, K., MANSSON, B. & NORIN, T. (1986). One short chemotherapy course given immediately after mastectomy: Experience of the SACSG. *INSERM*, **137**, 157.

NISSEN-MEYER, R., HOST, H., KJELLGREN, K., OTHERS (1987). Neoadjuvant chemotherapy in breast cancer – as single perioperative treatment and with supplementary long-term chemotherapy. *Adjuvant therapy of Cancer V*, Salmon, S.E. (ed), p. 253, Grune & Stratton Inc., Orlando, FLA.

NOLVADEX ADJUVANT TRIAL ORGANISATION (NATO) (1983). Controlled trial of tamoxifen as an adjuvant agent in management of early breast cancer. *Lancet*, **i**, 257.

NOLVADEX ADJUVANT TRIAL ORGANISATION (NATO) (1985). Controlled trial of tamoxifen as single adjuvant agent in management of early breast cancer. Analysis at six years. *Lancet*, **i**, 836.

PALSHOF, T. (1981). Adjuvant endocrine therapy in the management of primary breast cancer. *Rev. Endo-Related Cancer*, **7** (suppl.), 65.

PRITCHARD, K.I., MEAKIN, J.W., BOYD, N.F., OTHERS (1984). A randomized trial of adjuvant tamoxifen in postmenopausal women with axillary node positive breast cancer. *Proc. 4th International Conf on the Adjuvant Therapy of Cancer*, Tuscon, Jones, S.E. & Salmon, S.E. (eds), p. 339, Abs 49.

RIBEIRO, G. & PALMER M.K. (1983). Adjuvant tamoxifen for operable carcinoma of the breast: Report of clinical trial by the Christie Hospital and Holt Radium Institute. *Br. Med. J.*, **286**, 827.

ROSE, C., THORPE, S.M., MOURIDSEN, H.T., ANDERSEN, J.A., BRINCKER, H. & ANDERSEN, K.W. (1983). Anti-oestrogen treatment of postmenopausal women with high risk breast cnacer. *Breast Cancer Res. Treat.*, **3**, 77.

SCOTTISH CANCER TRIALS OFFICE (1987). Adjuvant tamoxifen in the management of operable breast cancer: The Scottish trial. *Lancet*, **ii**, 171.

TATE, H.C., RAWLINSON, J.B. & FREEDMAN, L.S. (1979). Randomized cooperative studies in the treatment of cancer in the United Kingdom: Room for improvement? *Lancet*, **ii**, 623.

WALLGREN, A., GLAS, U., THEVE, N.C., SKOOG, L. & MARDSJO, G. (1983). Adjuvant tamoxifen in operable breast cancer in Stockholm. *Proc, 13th International Congress of Chemotherapy, Vienna*, Spitzy, K.H. & Karrer, K. (eds), **18**, (271), 15.

WILSON, A.J., BAUM, H., BRINKLEY, D.M., DOSSETT, J.A., McPHERSON, K. & PATTERSON, J.S. (1985). Six-year results of a controlled trial of tamoxifen as single adjuvant agent in management of early breast cancer. *World J. Surg.*, **9**, 756.